# CS1 CAR-T targeting the distal domain of CS1 (SLAMF7) shows efficacy in high tumor burden myeloma model despite fratricide of CD8+CS1 expressing CAR-T cells

**DOI:** 10.1038/s41375-022-01559-4

**Published:** 2022-04-14

**Authors:** Julie O’Neal, Julie K. Ritchey, Matthew L. Cooper, Jessica Niswonger, L. Sofía González, Emily Street, Michael P. Rettig, Susan W. Gladney, Leah Gehrs, Ramzi Abboud, Julie L. Prior, Gabriel J. Haas, Reyka G. Jayasinghe, Li Ding, Armin Ghobadi, Ravi Vij, John F. DiPersio

**Affiliations:** 1 Department of Medicine, Washington University in Saint Louis, Saint Louis, MO 63110 USA; 2 Siteman Cancer Center, Washington University in St. Louis, St. Louis, MO 63110 USA; 3 Department of Radiology, Washington University in Saint Louis, Saint Louis, MO 63110 USA; 4 McDonnell Genome Institute, Washington University in St. Louis, St. Louis, MO 63108 USA; 5 Department of Genetics, Washington University in Saint Louis, Saint Louis, MO 63110 USA

**Keywords:** Immunotherapy, Myeloma

## Abstract

Despite improvement in treatment options for myeloma patients, including targeted immunotherapies, multiple myeloma remains a mostly incurable malignancy. High CS1 (SLAMF7) expression on myeloma cells and limited expression on normal cells makes it a promising target for CAR-T therapy. The CS1 protein has two extracellular domains – the distal Variable (V) domain and the proximal Constant 2 (C2) domain. We generated and tested CS1-CAR-T targeting the V domain of CS1 (Luc90-CS1-CAR-T) and demonstrated anti-myeloma killing in vitro and in vivo using two mouse models. Since fratricide of CD8 + cells occurred during production, we generated fratricide resistant CS1 deficient Luc90- CS1- CAR-T (ΔCS1-Luc90- CS1- CAR-T). This led to protection of CD8 + cells in the CAR-T cultures, but had no impact on efficacy. Our data demonstrate targeting the distal V domain of CS1 could be an effective CAR-T treatment for myeloma patients and deletion of CS1 in clinical production did not provide an added benefit using in vivo immunodeficient NSG preclinical models.

## Introduction

Multiple myeloma (MM), a malignancy of mature plasma cells, is the second most common blood cancer. Although expanded treatment options have improved patient outcomes [1], myeloma remains mostly incurable. The current lead MM CAR-T protein target is B-cell maturation antigen (BCMA) and although clinical response rates as high as 88% are described, the duration of response is only about one year [2–7] indicating new and second-generation strategies for CAR-T therapies in myeloma are needed.

Targeting alternative myeloma-expressed proteins with CAR-T therapy has the potential to improve patient outcomes. CS1 (SLAMF7, CRACC) is a rational target for myeloma CAR-T therapy since expression of CS1 is uniformly high on myeloma cells independent of cytogenetic abnormalities, genomic mutations, or disease stage [8, 9]. Expression within normal tissues is restricted to the hematopoietic system and includes mature NK cells, dendritic cells, plasma cells, and some T-cells. CS1 is not expressed on hematopoietic stem cells [8, 9]. Elotuzumab, a humanized CS1 antibody (Clone HuLuc63) that binds to the proximal C2 domain of CS1 is an effective anti-myeloma therapy when used in combination with lenalidomide and dexamethasone and without serious toxicity [10, 11] suggesting targeting CS1 with CAR-T will be effective and safe.

The CS1 protein has two extracellular domains useful for CAR targeting: the proximal Ig-like constant (C2) and the distal Ig-like variable (V) domain. CS1-CAR-T targeting the proximal C2 domain of CS1 (antibody clone HuLuc63 [8]); demonstrated anti-myeloma activity in preclinical models [12, 13] and is being pursued in clinical trials although results are not currently published. Targeting the distal C2 epitope of CS1 using (antibody clone Luc90, Luc90-CAR-T) provides an alternative strategy for CS1-CAR-T. Here, we report that Luc90-CAR-T demonstrate efficacy in a high tumor burden model of MM equivalent to HuLuc63-CAR-T. We observed self-killing (fratricide) of CD8-T-cells during Luc90-CAR-T production and show deletion of CS1 in T-cells (ΔCS1) protected a subset of CD8 T- cells from fratricide. Surprisingly, we found no survival benefit in ΔCS1-Luc90- CAR-T compared to Luc90-CAR-T even though the CD8 levels in the ΔCS1-Luc90- CAR-T were higher. These data suggest deletion of CS1 on Luc90-CAR-T would not be required for clinical production and that targeting the distal domain of CS1 may be effective in human myeloma patients.

## Materials and methods

### CAR-T design

Luc90 and HuLuc63 scFv (patent US8603477), CD19 scFv [14], CD79B scFv (patent US8691531B2) and BCMA (clone J22.xi, patent WO201406879A1) were synthesized (Genescript, Piscataway, NJ) and cloned into pLV (Vector Builder, Chicago, IL) or pELNS (kindly provided by Carl June, U of Penn).

### Lentivirus production

Lentivirus was produced using Calcium phosphate (Takara Bio, Mountain View, CA) or Lipofectamine (Invitrogen, Carlsbad, CA) as described [15].

### CAR-T Production

T cells were isolated from human PBMC from leftover platelet apheresis products or from purchased leukopacks (Miltenyi Biotech, Auburn, CA) using PAN-T kits and the AutoMACS (Miltenyi). CD4 and CD8 cells were purified separately using StraightFrom kits and a MultiMACS (Miltenyi). T cells were cultured as described [15]. Cells were activated with anti-CD3/CD28 dyna beads (Gibco, Waltham, MA) for 4–6 days or 2 days if guideRNA electroporation was performed.

### CRISPR/Cas9 deletion of CS1

The HGLibA_44319 guide sequence (underlined below; GACCAATCTGACATGCTGCA) was obtained from the GeCKO sgRNA human library (Addgene) modified as indicated and synthesized by either Trilink (San Diego CA) or IDT (Coralville, IA).

5ʹ2ʹOMe(G(ps)A(ps)C(ps))CAAUCUGACAUGCUGCAGUUUUAGAGCUAGAAAUAGCAAGUUAAAAUAAGGCUAGUCCGUUAUCAACUUGAAAAAGUGGCACCGAGUCGGUGC2′OMe(U(ps)U(ps)U(ps)U_3ʹ).

Electroporation was performed with buffer P3 (Lonza, Rockville,MD),15 μg spCas9 (Trilink Biotechnologies) and 20 μg gRNA on the 4D-Nucleofector (program EO-115) or with the same guides and 7.5 μg Cas9 and (10 μg guide) using MaxCyte ® buffer (Hyclone) and electroporated using the MaxCyte GT® system (Gaithersburg, MD).

### Cell lines

MM.1 S cells, kindly provided by Leif Bergsagel, were modified to MM.1S-CG [16]. ΔCS1-MM.1S-CGs were generated using the CS1 guide sequence above cloned into pMLM3636 (Addgene) lentiviral vector. MM1.S-CG cells were transduced with 1ug gRNA plasmid, 250 ng Cas9-HF plasmid and electroporated in SF solution using the Lonza 4D Nucleofector DS-137 program. CS1 negative cells were sorted using a MoFlow. OPM2 cells (DMSZ, Germany) were modified to express CBR-GFP as above.

### In vitro killing assays

CAR-T were incubated with targets at a range of (E:T) ratios. ^51^Chromium release assays were performed as described [17]. Bioluminescent killing assays were set up in a similar way. Killing was measured by Photon Flux using an AMI Imager (Spectral Instruments, Tucson, AZ).

### Flow cytometry

Antibodies used were CS1 (162.1), CD14 (M5E2), CD20 (2H7), CD19 (HIB19), IgG secondary, CD8 (RPA-T8), CD38 (BioLegend); CD4 (RPA-T4 or SK3) CD8 (RPA-T8), CD3 (UCHT1), CD56 (NCAM16.2), CD33 (WM53), CD45 (H130) BD Pharmingen; PE-CD34 Pool (Beckman Coulter), CD138, BCMA (Miltenyi), purified Luc90 (Creative Biolabs). 7AAD or Dapi was used for viability. Samples were run on an Attune or Yeti Flow Cytometer and analyzed using FlowJo V10 (TreeStar, Ashland, OR).

### Animal model and in vivo efficacy

Animal protocols compliant with regulations of Washington University School of Medicine Institutional animal care and use committee. Six to ten-week old NOD.Cg-*Prkdc*^*scid*^ *Il2rg*^*tm1Wjl*^/SzJ (NSG) male or female were used in all experiments. MM.1S-CG cells were injected intraveneously (i.v.) into tail veins of mice and treated with purified CAR-T cells i.v. For BLI, mice were injected intraperitoneally with 150 μg/g D-luciferin (Goldbio, Saint Louis, MO) and imaged as described [18] using an IVIS Imager (Perkin Elmer, Waltham MA) or an AMI Imager (Spectral Instruments, Tucson, AZ). Significant differences in survival were determined using Log Rank (Mantel-Cox) analysis.

## Results

### Luc-90-CAR-T demonstrates efficacy in a high tumor burden systemic myeloma model

**Fig. 1:**
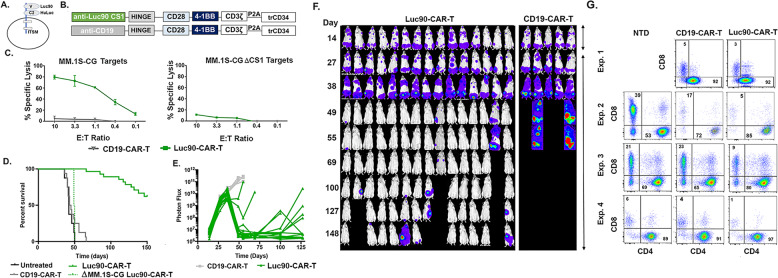
Luc-90-CAR-T demonstrates efficacy in a high tumor burden systemic myeloma model. **A** CS1 epitopes (**B**) CAR constructs (**C**) Luc90-CAR-T and CD19-CAR-T effectors were incubated with ^51^chromium labeled MM.1S-CG or MM.1S-ΔCS1 target cells for 4 h. Chromium release was used to assess killing. **D** Kaplan–Meier survival analysis of mice engrafted with either 5 × 10^5^ MM.1S-CG or MM.1S-CG-ΔCS1cells and treated with nothing, Luc90-CAR-T, or CD19-CAR-T. Median survival for mice treated with Luc90 CS1-CAR-T was not reached (*n* = 28), while median survival of CD19-CAR-T and untreated mice was 46 and 42 days, respectively (*n* = 13 each). Median survival of mice engrafted with MM.1S-CG ΔCS1 and treated with Luc90-CAR-T was 50 days (*n* = 2). **E**, **F** Representative bioluminescent imaging of mice. In (**E**), each line represents one mouse and in (**F**), normalized BLI images are shown. The mouse experiment is representative of four separate experiments (MM.1S-CG engrafted: CD19 CAR-T *n* = 16, Luc90 CS1 CAR-T *n* = 28, Untreated *n* = 8; MM.1S-CG ΔS1 CD19 CAR-T *n* = 4, Luc90 CS1-CAR-T *n* = 2). **G** Percentages of CAR + (CD34 + ) CD4 and CD8 in NTD, CD19-CAR-T and Luc90-CAR-T cultures.

CS1 has two extracellular epitopes useful for CAR-T targeting (Fig. 1A). CS1-CAR-T targeting the proximal C1 epitope (HuLuc63-CAR-T) showed activity in preclinical models [12, 13]. Here, we wanted to determine if targeting the distal V2 domain would provide an alternative option for CS1-CAR-T therapy. To this end, we designed a third generation CS1-CAR using the single chain variable fragment (scFv) from CS1 antibody clone Luc90 (Luc90-CAR-T; Fig. 1B). The CAR construct contained the scFv, a CD8 hinge, a CD28 transmembrane domain, CD28 and 4-1BB co-stimulatory domains and the CD3ζ signaling domain. A P2A self-cleaving peptide followed by a truncated human CD34 protein (trCD34) was incorporated to enable detection and purification of CAR-positive T cells (Fig. 1B).

To generate a matched negative control cell line for our studies, we used CRISPR/Cas9 technology to delete CS1 from the MM.1S-CG cell line (CS1 + and modified to express a green fluorescent -click beetle red luciferase fusion protein; CG) [16]. Knockout was confirmed using Western Blot and flow cytometry. Cell growth was not affected (Figure S1). We first tested killing activity of Luc90-CAR-T using ^51^chromium release assays. Luc90-CAR-T cells were incubated for 4 h with ^51^chromium- labeled MM.1S-CG or MM.1S-CG-ΔCS1 target cells at effector to target ratios (E:T) that ranged from 10:1 to 0.1:1. Luc90-CAR-Ts killed MM.1S-CG but not MM.1S-CG-ΔCS1 cells. CD19-CAR-Ts did not kill either cell line (Fig. 1C).

We tested in vivo efficacy of Luc90-CAR-T by injecting 0.5 × 10^6^ MM.1S-CG i.v. into the tail vein of NSG mice (day 0). When tumor burden was high (BLI signal 10^9^; day +28), mice were treated with 2 × 10^6^ Luc90-CAR-T (n = 28), CD19-CAR-T (*n* = 15) or were left untreated (*n* = 8). CAR-T cells were generated from four separate donors (Table S1). Luc90-CAR-T treated mice had a significant extension of survival compared to controls (*p* < 0.001; Fig. 1D). Mice engrafted with MM.1S-CG-ΔCS1 and treated with Luc90-CAR-T had similar survival to controls, confirming in vivo specificity (Fig. 1D). Bioluminescent imaging (BLI) demonstrated significant reduction of tumor with over half of the mice surviving long term tumor-free (Fig. 1E, F).

A subset of mice developed extramedullary tumors after Luc90-CAR-T treatment. Antigen escape is common after treatment with CD19-CAR-T [19] and is reported in myeloma patients treated with BCMA-CAR-T [2–4]. No clear loss of CS1 on excised tumors was observed (Fig. S2). We also found myeloma cells isolated from extramedullary tumors remained sensitive to Luc90-CAR-T cells in killing assays (Fig. S2), confirming antigen escape of CS1 was not common in our model and suggests the extramedullary tumors develop when cells migrate to sanctuary sites protected from CAR-T cells similar to a prior report [12].

Because CS1 is expressed on a subset of T-cells, we anticipated fratricide (or “self-killing”) of T-cells in our Luc90-CAR-T cultures. We assessed the CD4 and CD8 composition of Luc90-CAR-T and CD19-CAR-T cells and found the overall percent of CD8 + in Luc90-CAR-T cultures were consistently reduced compared to CD19-CAR-T (avg. 63% reduction; range 40-80%; Fig. 1G). This led to an increase in the percentage of CD4 + cells in Luc90-CAR-T-cells (avg. 89%, range 80-97) compared to CD19-CAR-T (mean 80%, range 65-92%; Fig. 1G). Even though fratricide occurred during Luc90-CAR-T production, Luc90-CAR-T composed of mostly CD4 + cells demonstrated efficacy in our mouse model of myeloma.

### Fratricide of CD8 CAR-T is prevented by CS1 deletion in T-cells

**Fig. 2:**
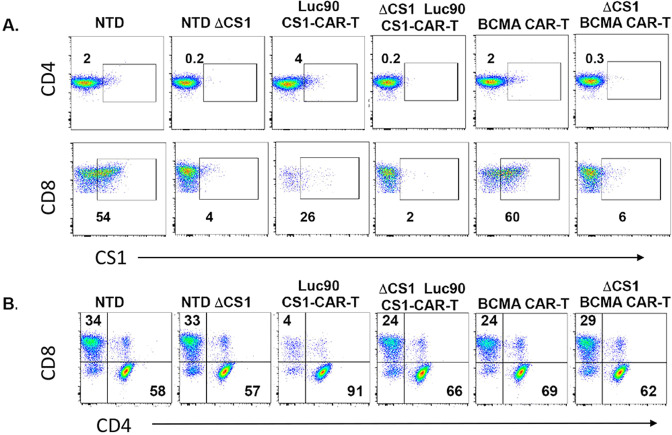
Fratricide of CD8 CAR-T is prevented by CS1 deletion in T-cells. **A** Flow cytometry was used to assess CS1 expression CD4 and CD8 cells on day +7 of CAR-T cultures as indicated. **B** Flow cytometry was used to assess percentage of CD4 and CD8 cells in CAR-T cultures. Representative experiment shown.

Although Luc90-CAR-T showed efficacy in vivo with a high CD4:CD8 ratio, it is well established that CD8 T-cells have cytotoxic properties that contribute to CAR killing efficacy. A 50:50 ratio of CD4:CD8 may provide the ideal T-cell ratio for CAR-T [20]. We hypothesized deletion of CS1 on T-cells prior to Luc90-CAR expression would prevent fratricide of CD8 + cells. To test this, we deleted CS1 in T-cells using CRISPR/Cas9 technology (“Methods”). We consistently observed greater than 90% deletion efficiency of CS1 on the surface of both CD4 and CD8 T-cells (Fig. 2A). We generated Luc90-CAR-T and ΔCS1-Luc90-CAR-T and found the ΔCS1-Luc90-CAR-T had CD8 levels comparable to control BCMA-CAR-T and ΔCS1-BCMA-CAR-T cultures (Fig. 2B). These data demonstrate feasibility of CS1 deletion in T-cells and protection of CD8 T-cells from fratricide in Luc90-CAR-T cultures.

We wanted to characterize CS1 expression in our CAR-T cultures to understand how CS1 expression changes over time with and without genetic deletion of CS1. To this end, we measured CS1 levels on CD4 and CD8 cells over a nine-day time course (Fig. S3). CS1 levels were higher on activated CD4 and CD8 cells compared to non-activated cells (Fig. S3). In non-transduced (NTD) T-cells, CS1 was expressed on about 30% of activated CD4 cells for the first four days of culture and then decreased to essentially undetectable levels. About 80% of activated CD8 T-cells expressed CS1 that decreased beginning on day +6 of culture (Fig. S3), consistent with a prior report [12]. It took three days for CS1 to become almost undetectable on the surface of CD4 T- cells and four days for this to occur on CD8 T-cells after genetic deletion of CS1 (Fig. S3).

### CS1 is not required for BCMA CAR-T efficacy

**Fig. 3:**
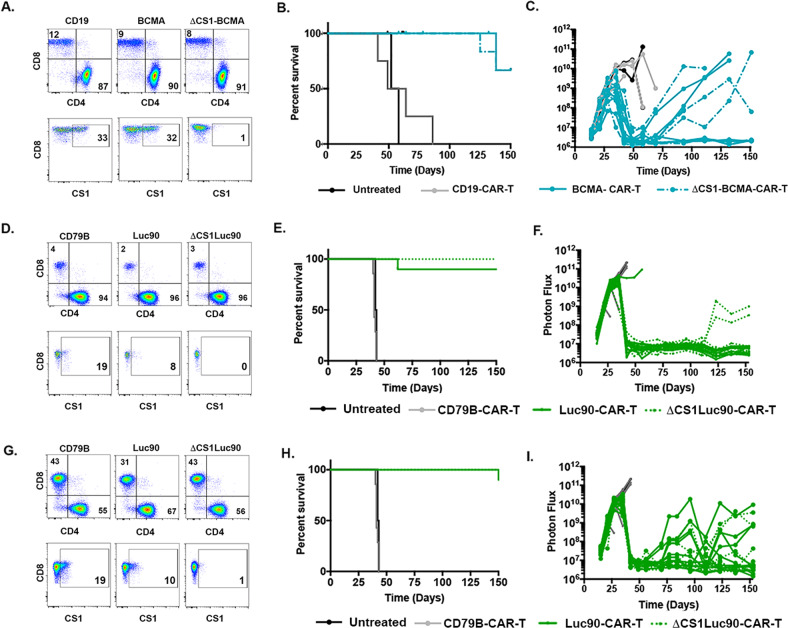
Efficacy Luc90-CAR-T is independent of CD4:CD8 ratio. **A** Flow cytometry was used to assess CD4 and CD8 percentages in CAR-T cultures as shown. Data shown are sorted CAR + cells **B** Kaplan–Meier Survival of mice treated with CD19-CAR-T (*n* = 4), BCMA CAR-T (*n* = 8, 4 were tumor free and censored due to infection at housing facility), ΔCS1-BCMA-CAR-T (*n* = 9, 3 were tumor free and censored due to infection at housing facility) or left untreated (*n* = 2). **C** Bioluminescent imaging. Each line represents one mouse. **D** Flow cytometry was used to assess CD4 and CD8 percentages in CAR-T cultures as shown that were designed to have a high CD4:CD8 ratio. Data shown are sorted CAR + cells. **E** Survival and (**F**) BLI of mice treated with Luc90-CAR-T (*n* = 10), ΔCS1 Luc90-CAR-T (*n* = 10), CD79B CAR-T (*n* = 3) and untreated mice (*n* = 2). **G** Flow cytometry showing CD4 and CD8 percentages of CAR-T as shown generated to have a lower CD4:CD8 ratio. **H** Survival and (**I**) tumor burden (BLI) in mice treated with Luc90-CAR-T (*n* = 10), ΔCS1 Luc90-CAR-T (*n* = 10), CD79B CAR-T (*n* = 3) and untreated mice (*n* = 2). Although graphed separately, survival plots show same control mice (CD79B and untreated) since experiment was performed at same time.

The functional role of CS1 in the CAR-T setting is not well described. To determine whether deletion of CS1 would be a viable option to improve efficacy of CS1-CAR-T, we wanted to determine if CS1 was required for CAR-T activity by comparing BCMA-CAR-T and ΔCS1-BCMA-CAR-T. We confirmed efficient deletion of CS1 ( > 97% assessed on CD8 cells) and observed the CD4:CD8 ratio was similar in both cultures (Fig. 3A). We predicted if CS1 was essential for CAR-T function, we would detect reduced survival and/or increased tumor burden in ΔCS1-BCMA-CAR-T treated mice compared to BCMA-CAR-T treated mice. No significant difference in survival or tumor burden was observed between the two groups demonstrating expression of CS1 was dispensable for BCMA-CAR-T function in our model (Fig. 3B, C). This suggested that deletion of CS1 in the setting of CS1-CAR-T would be an appropriate approach to compare efficacy of ΔCS1-Luc90-CAR-T to Luc90-CAR-T.

### Efficacy of Luc90-CS1-CAR-T is independent of CD4:CD8 ratio

To test our hypothesis that increased CD8 cells in our product would improve efficacy of Luc90-CAR-T, we compared survival and tumor burden of mice treated with Luc90-CAR-T or ΔCS1-Luc90-CAR-T using our high tumor burden MM.1S-CG mouse model, as above. T-cells isolated from three different donors were used to generate Luc90-CAR-T and ΔCS1-Luc90-CAR-T. Fratricide of CD8 T-cells occurred in the Luc90-CAR-T culture and protection of CD8 cells from death occurred in ΔCS1-Luc90-CAR-T cells (Fig. S4, Table S2). We observed similar survival and reduction of tumor burden in Luc90-CAR-T and ΔCS1-Luc90-CAR-T treated mice (Fig. S4). Although there was an average of a three-fold increase in the percentage of CD8 cells in ΔCS1-Luc90-CAR-T compared to Luc90-CAR-T, we noted the starting percentages of CD8 cells was relatively low (less than 25%; Table S2). This led to us to our next hypothesis that higher levels of CD8 T-cells in the starting cultures would lead to improved efficacy of ΔCS1-Luc90-CAR-T compared to Luc90-CAR-T.

To that end, we purified CD4 and CD8 T-cells separately from the same donor and plated them at two CD4:CD8 ratios. To mimic our prior experiment, we first plated T-cells with a high CD4:CD8 ratio (49:1 CD4:CD8 ratio; 98% CD4, 2% CD8). On day seven of culture control CD79B CAR-T were 4% CD8 + cells. Luc90-CAR-T were 2% CD8 + cells and ΔCS1- Luc-90-CAR-T were 3% CD8 cells (Fig. 3D). Deletion efficiency of CS1 assessed on CD8 cells was 97%. As expected, we observed similar survival and tumor burden of these CD8-low Luc90-CAR-T and ΔCS1- Luc-90-CAR-T in vivo (Fig. 3E, F) and similar to our previous experiment (Fig. S4). We next plated cells with a lower CD4:CD8 ratio (2.3:1CD4:CD8; 70% CD4; 30%CD8). On day 7, CD79B control CAR-T were 43% CD8 + cells Luc90-CAR-T were reduced to 31% CD8 and the ΔCS1-Luc90-CAR-T had 43% CD8 cells- similar to the CD79B controls, demonstrating protection of CD8 by deletion of CS1 (Fig. 3G). We found no significant survival difference between Luc90-CAR-T and ΔCS1-Luc90 CAR-T with the higher CD8 levels treated mice (Fig. 3H). We observed less tumor control in some of the mice in both groups at this CD4:CD8 ratio. Together, these data show that genetic deletion of CS1 to protect CD8 cells did not alter efficacy in our model.

### Targeting either extracellular epitope of CS1 by CS1-CAR-T is effective

**Fig. 4:**
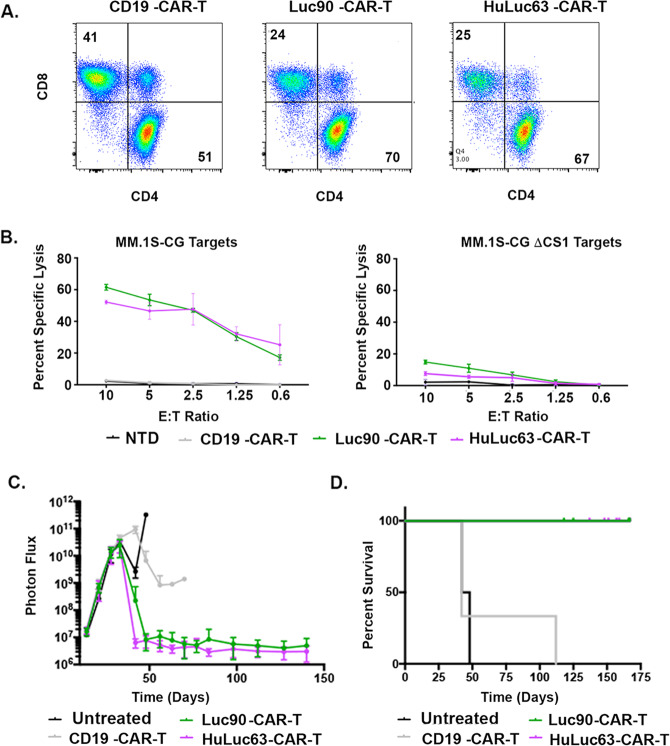
Targeting either extracellular epitope of CS1 by CS1-CAR-T is effective. **A** Flow cytometry of CD4 and CD8 populations. **B** Killing of MM.1S-CG targets by Luc90-CAR-T and HuLuc63-CAR-T assessed using ^51^chromium assays. **C** BLI of both CS1-CAR-Ts and (**D**) Kaplan–Meier survival analysis. Some mice were censored since they had no BLI signal but died of an infection.

Since CS1-CAR-T targeting the C2 proximal extracellular domain is being tested in clinical trials, we wanted to directly compare efficacy of HuLuc63-CAR-T and Luc90-CAR-T. Both caused fratricide of CD8 T-cells and induced similar killing of MM.1S-CG in vitro (Fig. 4A, B). We found similar survival and reduction of disease burden when we compared mice treated with HuLuc63-CAR-T and Luc90-CAR-T in our MM.1S-CG mouse model (Fig. 4C, S5). These data suggest CS1-CAR-T targeting either extracellular domain generates anti-myeloma activity in vitro and in vivo.

### 4-1BB only Luc90-CAR-T demonstrates anti-myeloma activity

**Fig. 5:**
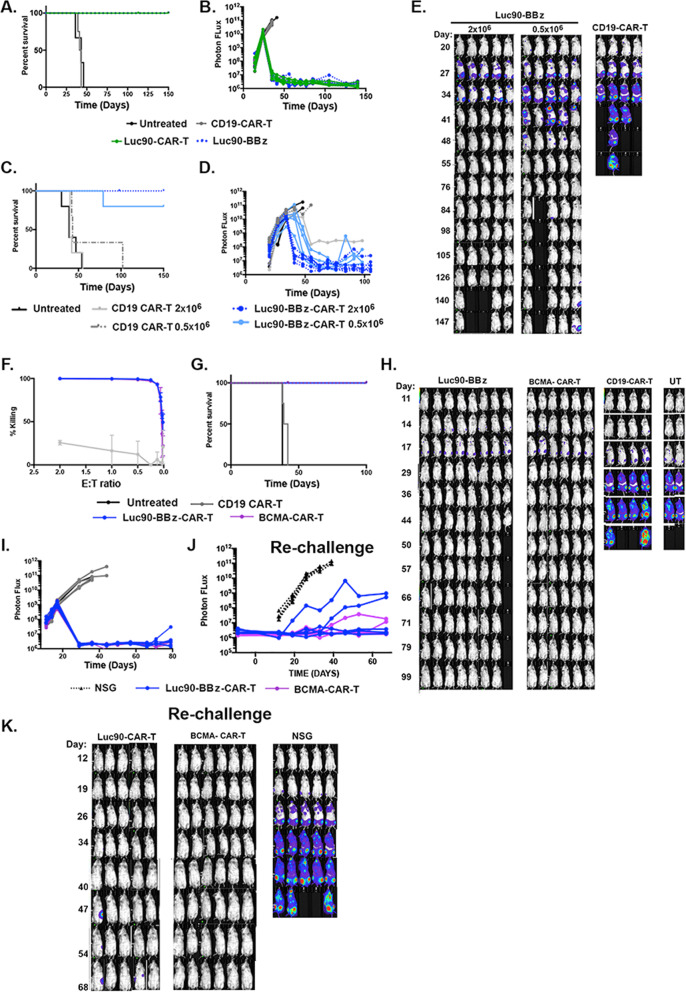
4-1BB only Luc90-CAR-T demonstrates anti-myeloma activity. **A** NSG mice were engrafted with MM.1S-CG cells and treated 24 days later with the original CD28/BBz Luc90-CAR-T (*n* = 5), Luc90-BBz-CAR-T (*n* = 8), CD19-CAR-T (*n* = 4) or were left untreated (*n* = 3) and monitored for survival. One Luc90-CAR-T mouse was censored due to cage flood. **B** Tumor burden measured by BLI. **C** NSG mice were engrafted with MM.1S-CG cells and treated 28 days later with 2 × 10^6^ or 0.5 × 10^6^ Luc90-BBz-CAR-T (*n* = 5), 2 × 10^6^ or 0.5 × 10^6^ CD19-CAR-T (*n* = 2 and 4, respectively) or left untreated (*n* = 5). Kaplan–Meier Survival shown. **D**, **E** Normalized BLI images shown. **F** In vitro killing of OPM2-CG cells after 48 h by Luc90-CAR-T and BCMA-CAR-T. **G** Mice were engrafted with 1 × 10^6^ OPM2-CG cells and treated 18 days later with 2 × 10^6^ Luc90-BBz-CAR-T or BCMA-CAR-T (CD28/BBz). Kaplan–Meier survival shown. **H**, **I** Longitudinal BLI was used to measure tumor burden. **J** Tumor free mice originally engrafted with OPM2-CG cells were re-challenged with 0.5 × 10^6^ MM.1S-CG cells. Five of seven Luc90-BBz-CAR-T were tumor free and seven of seven BCMA-CAR-T mice were included in the re-challenge experiment. Five tumor-naive NSG mice were used as controls. Tumor burden assessed by BLI is shown and normalized images shown in (**K**).

**Fig. 6:**
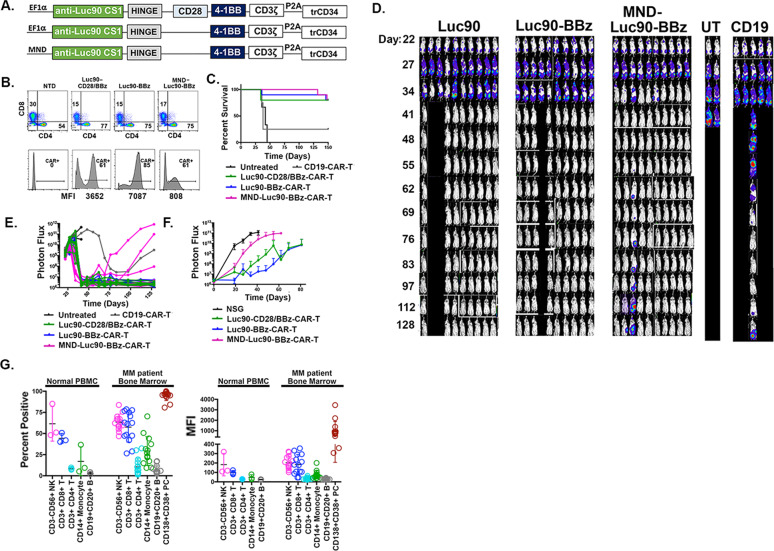
MND promoter drives lower CAR copy number but demonstrates efficacy in vivo. **A** CAR constructs. **B** Top row is flow cytometry of CD4 and CD8 cells as indicated. Bottom row are presorted CAR + cell percentages (assessed by flow using anti-CD34 antibody that binds to truncated CD34 that follows the P2A cleavage site). Below are the mean fluorescence intensities (MFI) of CAR + cells. **C** Kaplan–Meier survival analysis. **D** BLI and (**E**) Normalized images of the BLI. **F** Luc90-CAR-T (*n* = 7), Luc90-BBz (*n* = 7), MND-Luc90-CS1-CAR-T (*n* = 5) and NSG controls (*n* = 5) were re-challenged with 0.5 × 10^6^ MM.1S-CG. Longitudinal BLI shown. **G** Binding of Luc90 to normal hematopoietic and myeloma cells. Left: Percent of cells from normal PBMC or unsorted bone marrow myeloma samples that bound to Luc90 antibody using flow cytometry. Right: Mean fluorescence analysis demonstrating CS1 levels in cell subsets shown.

Our initial tests of Luc90-CAR-T used a construct containing both the CD28 and 4-1BB co-stimulatory domains. However, third generation CAR constructs are not currently widely used in the clinic. Since 4-1BB containing CAR-Ts generate clinical responses but with lower cytokine release syndrome (CRS) risk, and potentially longer persistence than CD28 containing CAR-T products, we wanted to confirm our prediction that a 4-1BB-only containing Luc90-CAR-T would a retain high anti-tumor activity. To this end, we engineered Luc90-CAR-Ts containing only the 4-1BB co-stimulatory domain (Luc90-BBz) and compared it to our original Luc90-CAR. We tested in vivo efficacy by engrafting NSG mice with 5 × 10^5^ MM.1S-CG cells and treating mice with 2 × 10^6^ CS1- Luc90-BBz CAR-T, Luc90-CAR-T or controls. No significant differences in survival or tumor burden were observed in two separate experiments (Fig. 5A, B and Fig. 6C–E below).

We next tested whether a lower dose of Luc90-BBz CAR-T would control tumor burden in our model. We treated MM.1S-CG engrafted mice with the standard 2 × 10^6^ or a lower dose of 5 × 10^5^ Luc90-BBz -CAR-T. Although the kinetics were slightly delayed compared to mice treated with 2 × 10^6^ Luc90-BBz-CAR-T, the mice treated with the lower dose had a significant survival benefit and tumor reduction compared to controls (Fig. 5C–E). Since 4-1BB is the costimulatory domain we would prioritize for clinical use, we wanted to further assess Luc90-BBz function by using the OPM2 cell line (CS1 + and modified to express CBR-GFP; “Methods”) as target cells. Both Luc90-BBz and BCMA-CAR-T (CD28/BBz) showed potent in vitro killing activity (Fig. 5F). We engrafted NSG mice with 2 × 10^6^ OPM2-CG cells and treated mice eighteen days later (BLI signal: 10^9^) with 2 × 10^6^ Luc90-BBz CAR-T, BCMA-CAR-T or controls. Luc90-BBz and BCMA-CAR-T treated mice had significant extension of survival and reduced tumor burden compared to CD19-CAR-T (Fig. 5G–I). We assessed the function of long-lived Luc90-BBz and BCMA-CAR-T in this model by re-challenging tumor free mice with 0.5 × 10^6^ MM.1S-CG cells. Compared to NSG control mice, both Luc90-BBz and the BCMA CAR-T controlled tumor growth (Fig. 5J, K) demonstrating high activity of Luc90-BBz-CAR-T.

### MND promoter drives lower CAR copy number but demonstrates efficacy in vivo

As we consider Phase I clinical testing of Luc90-BBz CAR-T in myeloma, we collaborated with VIVEBiotech to make clinical grade lentivirus for use in preclinical testing and for eventual clinical trials in patients with relapsed and refractory multiple myeloma. In all of the studies presented so far, CAR expression was driven by the EF1α promoter since it drives efficient CAR expression in human T-cells [21]. However, VIVEBiotech found viral titers were seven times higher with MND-containing promoters compared to EF1α (not shown).This phenomenon has recently been reported by a separate group [22]. Since MND is being used clinically in an effective FDA approved product (BCMA-CAR-T, idecabtagene vicleucel [23]) and higher titer virus would enable treatment of more patients, we sought to assess efficacy of MND-Luc90-BBz CAR-T (MND-Luc90-CAR-T); (Fig. 6A). To this end, we generated both the original third generation Luc90-CAR-T, Luc90-BBz-CAR-T and compared them to MND-Luc90-BBz CAR-T in our MM.1S-CG mouse model, as above. Survival of mice was similar across groups (Fig. 6C) as was tumor control assessed by BLI (Fig. 6D, E). Three of ten MND-Luc90-BBz mice had increased tumor burden over time while the other seven remained tumor free for the duration of the experiment. Similar results were observed in two separate repeat experiments (Fig. S6). We assessed long-term function of the Luc90-CAR-Ts by re-challenging mice with 0.5 × 10^6^ MM.1S-CG cells and found reduced tumor burden in all three Luc90-CAR-T groups indicating long-lived CAR-T cells were present in all mice. In the first experiment, the MND-Luc90-BBz re-challenged mice had inferior tumor control compared to both Ef1α promoter containing CAR-Ts (Fig. 6) but that was not true in the repeat experiment (Fig. S7). Together, these data demonstrate efficacy of MND- promoter driven CS1-CAR-T in both primary and re-challenge setting.

A recent report showed the mean fluorescence intensity (MFI) of CAR expression was lower in MND vs Ef1α promoter containing constructs, suggesting MND is a weaker promoter than EF1α leading to lower copies of CAR on the surface of the T-cells (22). To assess if this was true for our CS1 CAR-Ts, we quantitated MFI of CAR + cells in our studies. We found lower MFI signal in MND-Luc90BBz-CAR-T compared to Luc90-CAR-T even when the transduction efficiency of MND-Luc90BBz was higher. This occurred in CAR-Ts generated from multiple donors (Fig. 6B, Fig. S6). Although the expression of CS1 CAR driven by the MND promoter was lower, we did observe significant efficacy in our high tumor burden mouse model in both primary tumor models and in the context of re-challenge. An advantage of lower expression raises the possibility of finding a balance of efficacy and safety. Future experiments will determine if the MND containing CS1-CAR-T has a reduced potential for causing CRS in models we developed in our lab.

Luc90 has never been tested in human clinical trials as an immunotherapy, so we sought to assess the potential ‘on target- off tumor’ toxicity of Luc90. To this end, we assessed binding of Luc90 to immune cells in PBMCs isolated from three healthy donors and unsorted bone marrow samples from fourteen myeloma patients using flow cytometry. Luc90 bound to the highest percentage of CD3-CD56 + NK and CD3 + CD8 + T-cells. A much smaller percentage of CD3 + CD4 + T-cells, CD14 + monocytes and CD19 + CD20 + B cells bound to Luc90 (Fig. 6G, Fig. S8). These results are consistent with our data showing fratricide of CD8 T cells by Luc90-CAR-T cells and with flow cytometry using a commercially available CS1 antibody (clone 162.1) that showed binding of CS1 to CD8 T-cells, NK, and binding to lower percentages of monocytes, CD4 T cells and B cells (Fig. 6G, Fig. S8; data not shown) and previously reported [12]. Since Luc90-CAR-T shows potent anti-myeloma activity against OPM2-CG and MM.1 S cell lines that endogenously express CS1, we predicted it would efficiently bind to human myeloma cells. As expected, Luc90 bound to most CD138 + CD38 + MM cells (Fig. 6G, Fig. S8). Using mean fluorescence intensity (MFI) analysis, we found the MFI levels for Luc90 in MM cells was almost always higher on MM cells when compared to normal cells.

## Discussion

Here, we report efficacy of Luc90-CAR-T targeting the distal V2 domain of CS1 in a high tumor burden mouse model of myeloma. Luc90-CAR-T reduced tumor burden and significantly extended survival of tumor bearing mice compared to controls when tested in both a third generation (CD28/4-1BB) and a 4-1BB only second-generation format. A prior report demonstrated higher efficacy of Luc90-CD28-CAR-T compared to Luc90-BBz CAR-T [24]. We did not observe an efficacy difference between Luc90-BBz or Luc90-CD28 (not shown) and here chose to focus solely on 4-1BB containing CARs for safety reasons.

It is possible the efficacy of targeting each CS1 epitope with CS1-CAR-T will not be the same. We acknowledge we are not the first to test CS1-CAR-T and in our studies, we found similar efficacy of Luc90-CAR-T and HuLuc63-CAR-T, currently being tested clinically although without published results. A prior study reported HuLuc63 CS1-CAR-T had higher efficacy than Luc90-CAR-T [12]. The differences observed between our data and both of those studies could be explained by differences in study design. Prior studies show cell surface proximal epitopes on CAR target proteins facilitate efficient contact to CAR-T which is required for signaling and function of CAR-T cells [25]. Although this might predict targeting the distal V domain (Luc90) would prove inferior to targeting the proximal C (HuLuc63) domain, we observed similar efficacy of both CS1 CAR-Ts. The small size (24 kDa) of the CS1 extracellular domain likely explains why both are effective. However, updated data presented at the American Society of Hematology indicates BCMA-CAR-T (cilta-cel, Janssen) targeting two BCMA domains shows higher response rates and durability than ide-cel (bb2121) targeting one domain (both with the 4-1BB co-stimulatory domain) [26, 27]. These data suggest that clinical trials are needed to assess efficacy of proximal vs distal CS1-CAR-T targeting. Additionally, herein, we tested multiple Luc90-CAR constructs by making changes to the promoter, co-stimulatory domain, CD4:CD8 ratio and deleting CS1. None of these changes dramatically affected our pre-clinical efficacy results. However, it must be noted that in human studies, these changes could potentially have significant effects on efficacy and safety.

There remains an ‘off-tumor and on-target’ limitation of targeting CS1 on hematopoietic cells: the potential for inducing a state of prolonged immunodeficiency due to high CS1 expression on CD8 T, NK, and probably also invariant NKT cells (iNKT) that may be clinically consequential. Since Luc90 has not yet been tested in human clinical trials we addressed this by assessing binding of Luc90 to cell subsets in PBMC isolated from healthy donors and unsorted bone marrow from myeloma patients. Luc90 bound to CD3-CD56 + NK and CD3 + CD8 + T-cells as expected, consistent with flow cytometry data using the CS1 162.1 antibody in a similar analysis and also data demonstrating that HuLuc63-CAR-T was toxic to primary CD8 T-cells and NK cells in an in vitro killing assay [12]. Although Luc90 and HuLuc63 CAR-Ts demonstrate similar anti-tumor effects and some overlapping ‘on target-off tumor’ binding, some of the observed toxicities seen with all CAR-T products (CRS and neurotoxicity) may be significantly different when tested in man. This can only be determined in the context of ongoing and future clinical trials and may help us determine if additional safety measures such as suicide genes are required to prevent this potential complication.

Targeting CS1 in the CAR-T setting is unique compared to other CAR targets. Most CAR-T protein targets are not expressed on T-cells (i.e. CD19, BCMA) so fratricide is not an issue. T-cell malignancies represent the other extreme, where all/most T-cells express the target, as does the tumor (i.e. CD3, and CD7) and therefore target deletion is essential [28, 29]. We and others have shown that CS1-CAR-T has potent anti-myeloma efficacy even in the presence of fratricide of CD8 cells. We hypothesized protecting CD8 cells from fratricide by genetically deleting CS1 would improve efficacy of CS1-CAR-T in myeloma however, using multiple methods to test this, we saw neither a decrease nor an increase in efficacy in ΔCS1-Luc90-CS1-CAR-T compared to Luc90-CS1-CAR-T.

Herein, we tested CAR-T comprised of mostly CD4 T-cells. This occurred because (1) multiple donors had few CD8 cells to start, (2) fratricide further reduced the percentage of CD8 cells and (3) we intentionally generated a mostly CD4 CS1-CAR-T product by controlling the CD4:CD8 ratios. In all situations, we observed significant efficacy of CS1-CAR-T. These data suggest that CS1-CAR-T activity is not critically dependent on a high percentage of CD8 cells and clinical production would not require genetic deletion of CS1. Testing CS1-CAR-T in immunocompetent mouse models and human clinical trials will determine the efficacy of high CD4:CD8 ratio CS1-CAR-T. The CARAMBA trial protocol [30] is designed to control the CD4:CD8 ratio; the results of this study compared to other studies not actively modulating CD4:CD8 ratio should provide insight to this issue.

CS1 is a Type I glycoprotein cell surface receptor and a member of the signaling lymphocyte activation molecule (SLAM) family [31]. It is a positive regulator of NK function mediated by the adaptor protein EAT-2 [32]. An inhibitory function for CS1 is reported in mouse CD4 T- cells [32] however, the role of CS1 in human T (or CAR-T) cells is not well defined. Our data showing equivalent in vivo efficacy of BCMA-CAR-T and ΔCS1-BCMA-CAR-T suggests deletion of CS1 does not negatively impact CAR mediated killing. The upregulation of CS1 on T-cells upon anti-CD3/CD28 activation (Fig S4) suggests CS1 might play a role in activation of T-cells. It took two to four days for the loss of surface CS1 to occur after guide RNA delivery (Fig S4) so CS1 expression was intact during initial T-cell activation of T-cells. Future experiments to characterize expression of adapter proteins EAT-2 and SAP and deletion of CS1 prior to activation will help characterize the role for CS1 in activation of human T-cells.

Although CS1 (in addition to BCMA) represents an attractive primary target in patients with relapsed and refractory myeloma due to its highly regulated and consistent overexpression in myeloma cells, other identified targets may be clinically important, especially for patients who progress after BCMA-CAR-T or BCMA-bispecific therapies. Several of these have entered clinical trials and include CD38, CD138, CD19, GPRC_5_D and FCRL5 [33, 34]. Although their efficacy vs. toxicities need to be assessed in clinical trials, the gene and protein expression profiles of some of these potential targets using publicly available datasets suggest significant expression in off-target tissues such as the GI tract (Figure S7). Comprehensive analyses of other differentially expressed myeloma surface and intracellular targets are underway by many groups which may yield future targets for CAR-T, bispecific and TCR-T based therapies.

## Supplementary information

Supplemental Information
