# Supplementary material for: CS1 CAR-T targeting the distal domain of CS1 (SLAMF7) shows efficacy in high tumor burden myeloma model despite fratricide of CD8+CS1 expressing CAR-T cells
Source: Leukemia. 2022 Apr 14;36(6):1625–34. doi: 10.1038/s41375-022-01559-4 (PMC9162922; doi:10.1038/s41375-022-01559-4)
Supplement: Supplementary file 1 — Supplemental Information [file 41375_2022_1559_MOESM1_ESM.pdf]

Supplemental Information

Figure S1. Generation of MM.1S cells deficient for CS1 protein A. Western blot analysis of MM.1S cells, MM.1S -CG, MM.1S ∆CS1-CG cells and Jurkat cells (CS1-) confirming deletion of CS1 protein in MM.1S ∆CS1-CG cells. **B**. Flow cytometry was also used to confirm deletion of CS1 in MM.1S ∆CS1-CG cells. **C.** 100,000 cells/well were plated in triplicate and cell counts were performed for ten days in culture. Data are representative of two independent experiments. **D**. Mice were engrafted with 500,000 MM.1S-CG or MM.1S ∆CS1-CG cells. BLI on day eighteen confirms tumor cells localized to same sites in MM.1S-CG and MM.1S ∆CS1-CG engrafted mice. BLI (each mouse is represented by an individual line) demonstrates similar tumor burden over time.

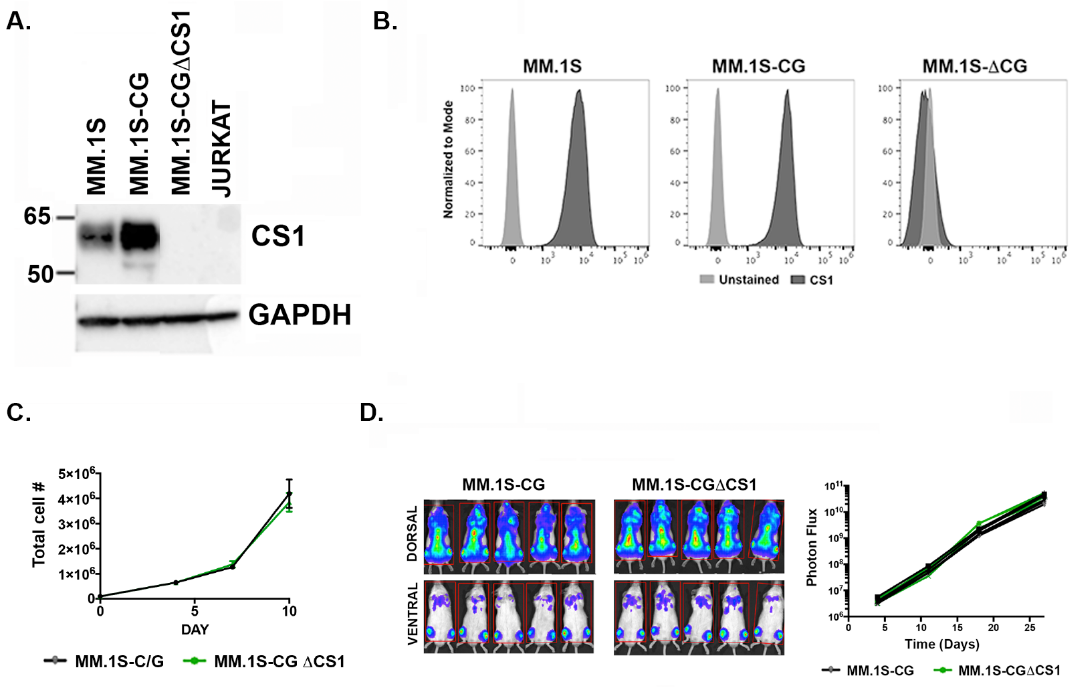

Figure S2. A. Antigen escape not common in extramedullary tumors isolated from Luc90-CAR-T treated mice Flow cytometry was used to assess CS1 expression on MM.1S-CG tumor cells excised from mice. Here, we compared expression of the parental MM.1S-CG cells to GFP+ MM.1S-CG cells isolated from the bone marrow of mice treated with CD19-CAR-T, where there is no selective pressure to lose CS1 expression. Excised tumor cells from CD19-CAR-T showed slightly lower expression than the cultured cell line. This was compared to an extramedullary tumor isolated from a Luc90-CAR-T treated mouse. MM.1S ΔCS1-CG were used a negative control for flow. Representative data shown **B**. Three separate extramedullary tumors were isolated from mice that developed a tumor after treatment with Luc90-CAR-T and used as targets in chromium killing assays. Luc90-CAR-T killed MM.1S-CG tumor cells while CD19-CAR-T did not.

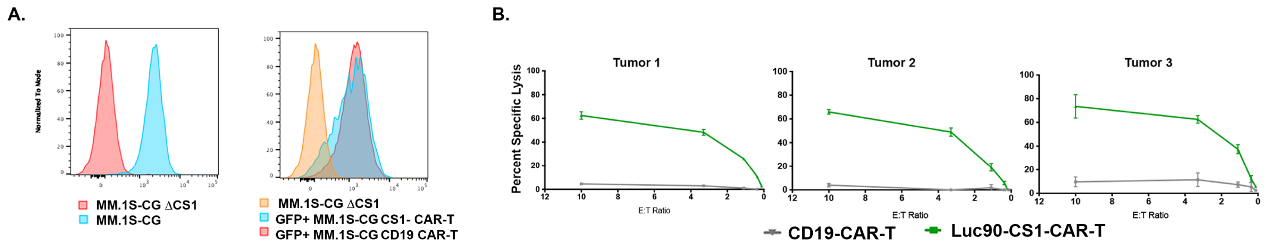

Figure S3. Longitudinal tracking of CS1 expression in CAR-T cultures A. CS1 in CD4 and CD8 cells day 0-2 **B**. CS1 on CD4 cells and **C**. CD8 cells (top) and bottom percentages of CD4 and CD8 on day 7 of culture.

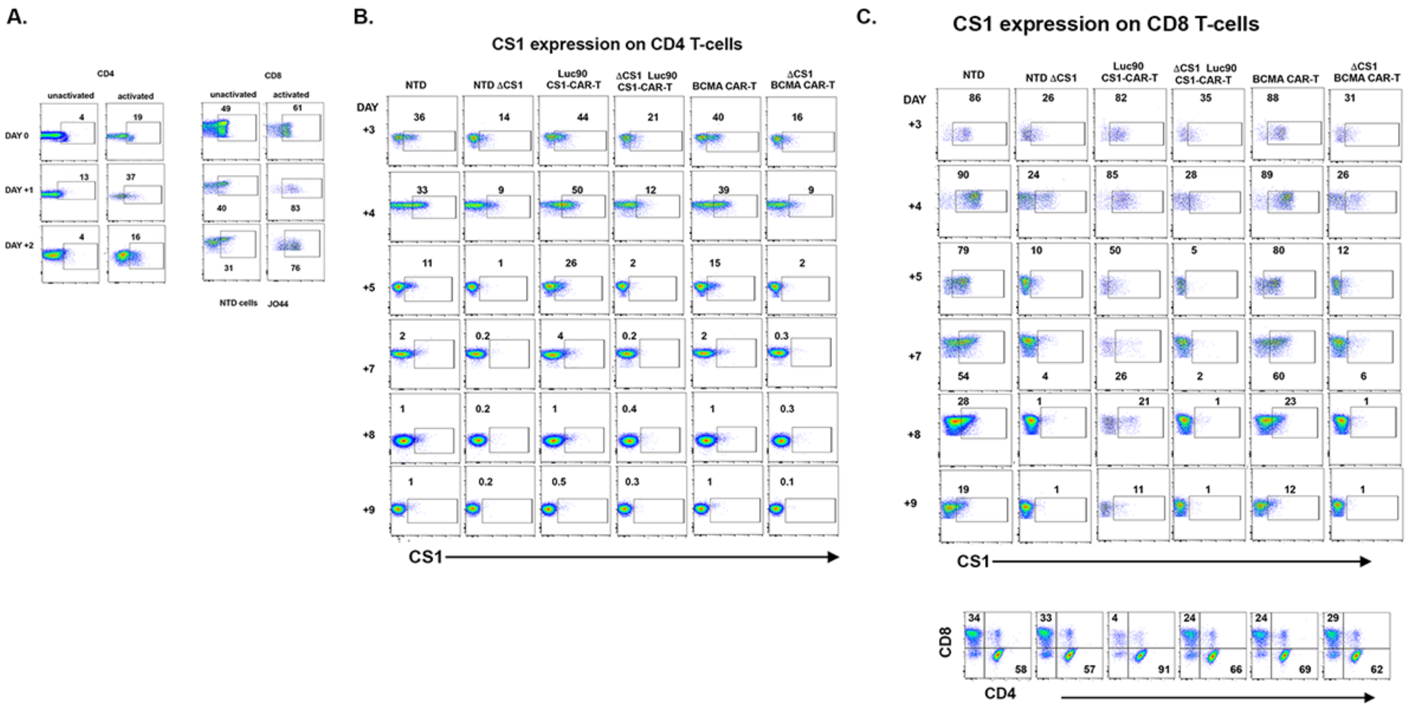

Figure S4. **Similar efficacy of Luc90-CAR-T and ∆CS1-Luc90-CAR-T A**. Flow cytometry showing CD4 and CD8 percentages in CAR-T cultures from three separate donors All plots are post CD34 selection of CAR positive cells and T-cells here were isolated using PAN-T negative selection kits so the CD4 and CD8 cells are collected together. **B**. Top: Kaplan Meier data from three separate experiments. 5X10^6 cells were engrafted i.v. into tail veins of mice and 28 days later when tumor burden was high, mice were treated with Luc90-CS1-CAR-T, ∆CS1-Luc90-CAR-T, either a CD79B-CAR-T or CD19-CAR-T as a negative control or were left untreated. Bottom: BLI of mice from each experiment. Each line represents one mouse. Data represent three separate experiments. Untreated n=8; Control CAR-T (CD19 or CD79B, n=13); Luc90 CS1-CAR-T n=21; ∆CS1 Luc90 CS1-CAR-T n= 17. Luc90-CS1 CAR-T.

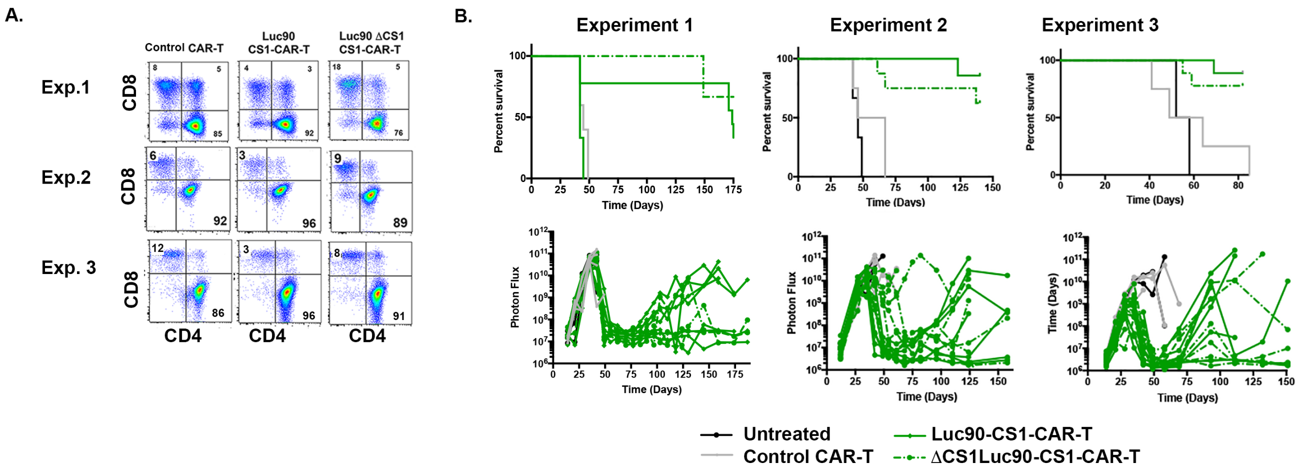

Figure S5. Similar efficacy of Luc90-CAR-T and HuLuc63-CAR-T A. Flow cytometry of CD4 and CD8 cells in CAR-T cultures as shown. **B**. Chromium release killing assays comparing Luc90-CAR-T and HuLuc63 CAR-T. MM.1S-CG cells (left) or MM.1S-CG ΔCS1 (right) were used as target cells **C**. Mice were treated Kaplan Meier analysis comparing survival of mice treated with 2X10^6 Luc90-CAR-T, HuLuc63-CAR-T or controls. **D**. BLI of mice shown in **C**. For this study T-cells were activated with anti-CD3/CD8 matrix Transact Miltenyi Inc, Auburn, CA and grown in TexMACS media (Miltenyi Inc, Auburn, CA) supplemented with 10ng/ml IL-7 and 10ng/ml IL-15.

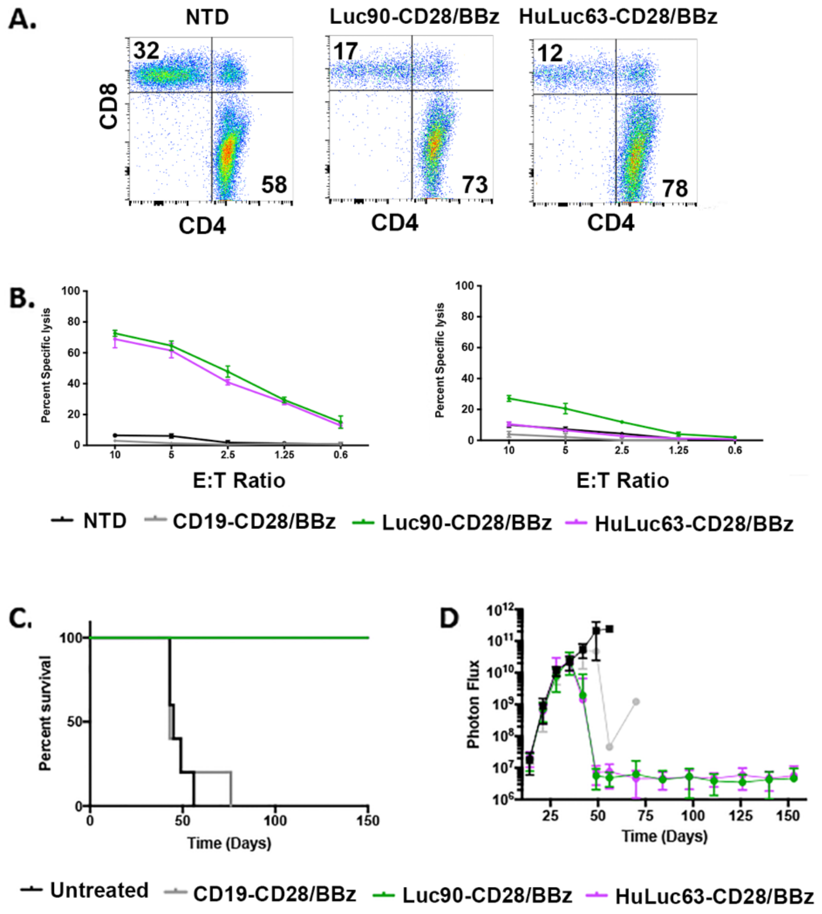

Figure S6. Efficacy of EF1α and MND CS1-CAR-Ts A. Top: Flow cytometry of CD4 and CD8 cells of CAR-T as labeled. Bottom: Percent CAR+ shown and MFI of CAR+ cells shown below the plots (assessed by CD34). **B**. 5X10^6 cells were engrafted i.v. into tail veins of mice and treated 28 days later. Kaplan Meier Survival shown. One Luc90-CAR-T mouse was censored due to a cage flood but was tumor free. **C**. BLI of mice treated with 2X10^6 Luc90-CAR-T (n=5), Luc90-BBz -CAR-T (n=8), MND-Luc90-BBz (n=5), CD19 CAR-T (n=4) and Untreated mice (n=3). A separate repeat experiment is shown in **D-H**. **D**. Top: Flow cytometry of CD4 and CD8 cells of CAR-T as labeled. Bottom: Percent CAR+ shown and MFI of CAR+ cells shown below the plots (assessed by CD34). **E**. Mice were engrafted with MM.1S-CG and treated with 2X10^6 Luc90-BBz (n=5), MND-Luc90 CAR-T (n=3) or CD19-CAR-T (n=4) as above. **E**. Kaplan Meier survival **F**. BLI **G**. Normalized BLI images **H**. Luc90-BBz (n=4), MND-Luc90 CAR-T (n=3) and NSG controls (n=5) were re-challenged with 0.5X10^6 MM.1S-CG. Longitudinal BLI shown. One Luc90-BBz mouse was censored due to a swollen face but was tumor free.

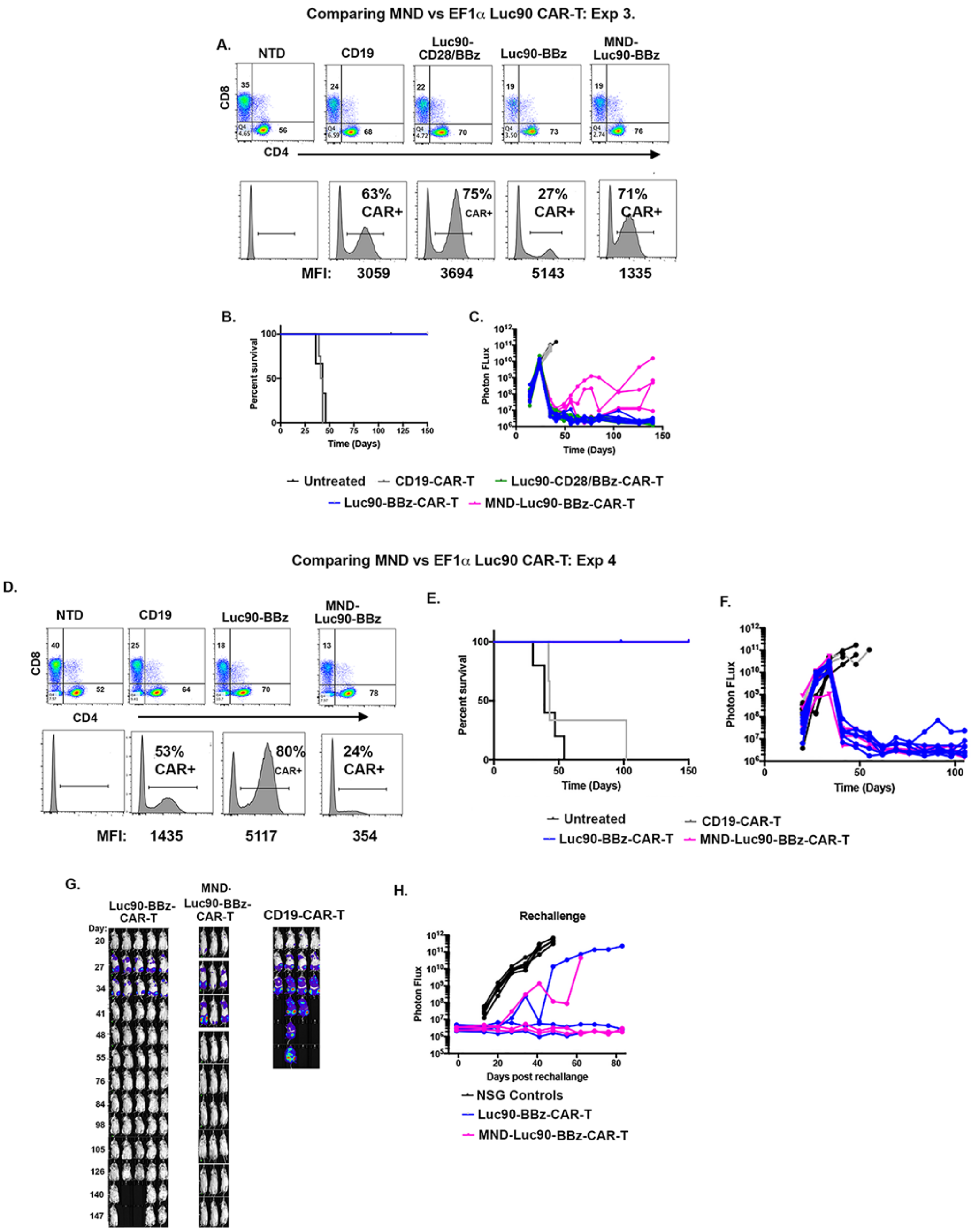

Figure S7. A. **Expression of CD19, CD38, FCRL5 and GPRC5D across normal tissues**. **A**. Expression profiles for a subset of proteins in human tissues based on immunohistochemistry from the human protein atlas (https://www.proteinatlas.org/about/download). For each gene, tissues are labeled on the y axis and level of detection (High, Medium, Low, Not detected) is indicated on the x axis. A black dot indicates the expression level for each gene. **B**. Consensus transcript expression levels from the human protein atlas. For each tissue (y-axis) and gene (x-axis), the normalized expression (NX) based on data from three sources is plotted. For genes with NX greater than 25 their value is indicated by a black dot of the same indicated size. For genes with NX less than 25 the normalized expression is indicated by size of the dot and color indicated in the legend.

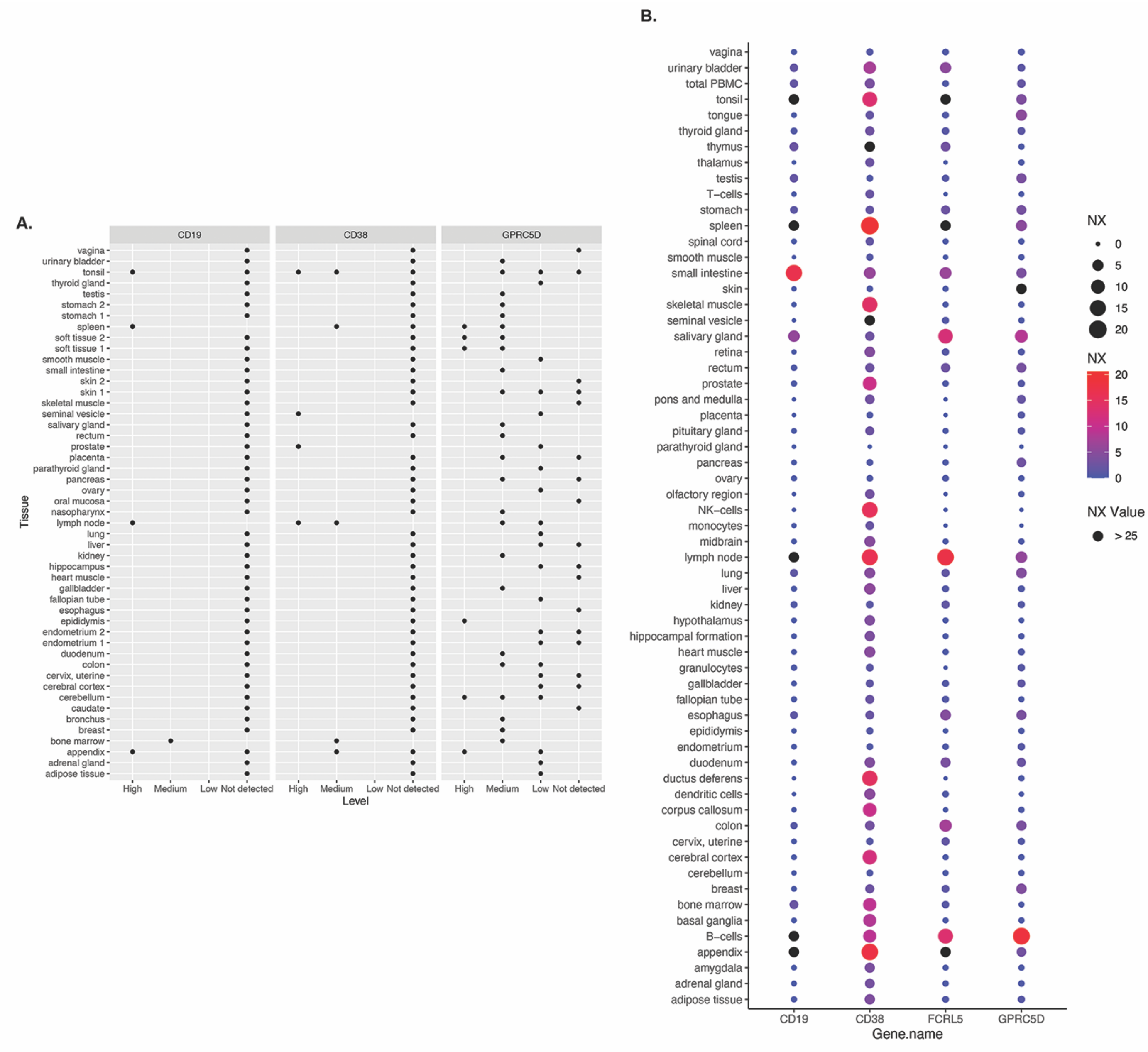

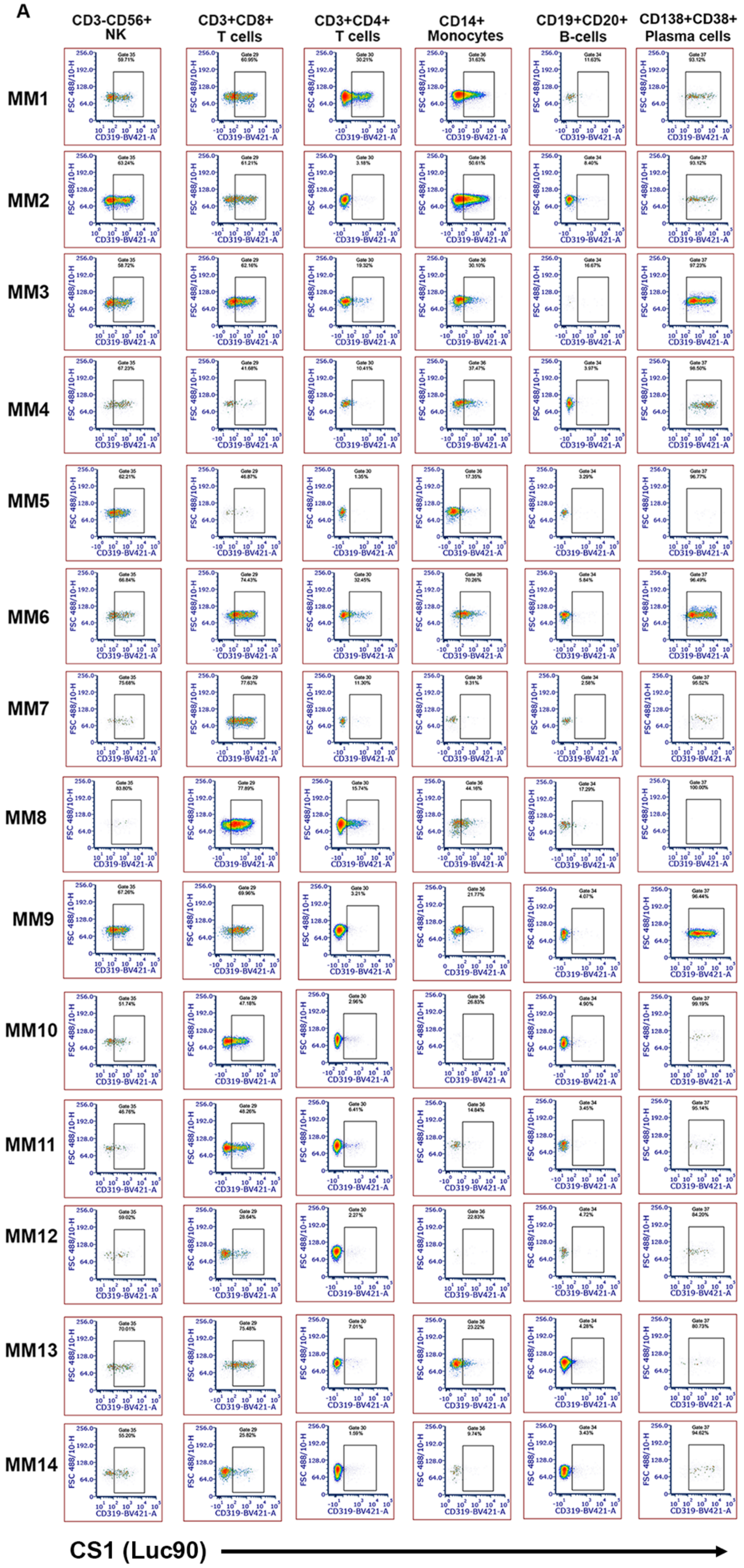

Figure S8. Binding of Luc90 to immune cells A. Flow cytometry plots of Luc90 binding to immune cells in unsorted bone marrow isolated from fourteen myeloma patients **B.** Similar flow cytometry as in A but here on PBMC isolated from three healthy donors.

B.

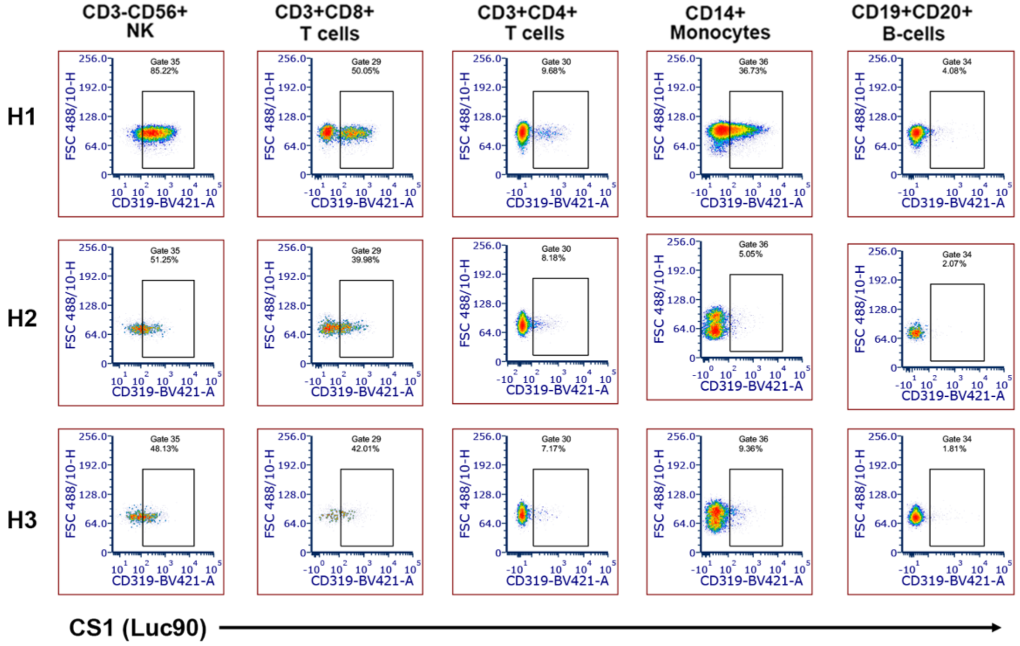

CD3-CD56+ NK
CD3+CD8+ T cells
CD3+CD4+ T cells
CD14+ Monocytes
CD19+CD20+ B-cells
H1
H2
H3
FSC 488/10-H
CD319-BV421-A
Gate 35 85.22%
Gate 29 50.05%
Gate 30 9.68%
Gate 36 36.73%
Gate 34 4.08%
Gate 35 51.25%
Gate 29 39.98%
Gate 30 8.18%
Gate 36 5.05%
Gate 34 2.07%
Gate 35 48.13%
Gate 29 42.01%
Gate 30 7.17%
Gate 36 9.36%
Gate 34 1.81%
CS1 (Luc90)

Table S1. Distribution of mice used for efficacy studies in mouse experiments shown in Figure 1.

Sample	Mouse numbers				
	Exp. 1	Exp. 2	Exp.3	Exp.4	Total
CD19 CAR-T	4	4	3	5	16
Luc90 CS1 CAR-T	5	3	5	15	28
Untreated	3	5	0	0	8
MM.1S-CG ΔCS1 Luc90 CS1 CAR-T	2				2
MM.1S-CG ΔCS1 CD19 CAR-T	4				4
Total	18				

Table S2. Mouse numbers and %CD8 cells in Luc90 vs ΔLuc90 cultures for data shown in Figure S4.

	Mouse numbers			
Sample	Exp. 1	Exp. 2	Exp.3	Total
CD19 or 79B CAR-T	5	4	4	13
Luc90 CS1 CAR-T	9	6	6	21
ΔCS1 Luc90 CS1 CAR-T	3	7	7	17
Untreated	5	3	2	10

	Percent CD8 cells in CAR-T cultures			
Sample	Exp. 1	Exp. 2	Exp.3	Average
NTD	18	25	19	21
CD19 or 79B CAR-T	8	6	12	9
Luc90 CS1 CAR-T	4	3	3	3
ΔCS1 Luc90 CS1 CAR-T	18	9	8	12
